# A New Synergistic Strategy for Virus and Bacteria Eradication: Towards Universal Disinfectants

**DOI:** 10.3390/pharmaceutics14122791

**Published:** 2022-12-13

**Authors:** Loïc Leclercq, Véronique Nardello-Rataj

**Affiliations:** UMR 8181-UCCS-Unité de Catalyse et Chimie du Solide, Université de Lille, CNRS, Centrale Lille, Université d’Artois, 59000 Lille, France

**Keywords:** didecyldimethylammonium chloride, *N*,*N*-bis(3-aminopropyl)dodecylamine, cyclodextrin, bactericide, virucide, poliovirus, norovirus, *Pseudomonas aeruginosa*, synergistic formulation

## Abstract

In response to the COVID-19 and monkeypox outbreaks, we present the development of a universal disinfectant to avoid the spread of infectious viral diseases through contact with contaminated surfaces. The sanitizer, based on didecyldimethylammonium chloride (DDAC), *N*,*N*-bis(3-aminopropyl)dodecylamine (APDA) and γ-cyclodextrin (γ-CD), shows synergistic effects against non-enveloped viruses (poliovirus type 1 and murine norovirus) according to the EN 14476 standard (≥99.99% reduction of virus titer). When a disinfectant product is effective against them, it can be considered that it will be effective against all types of viruses, including enveloped viruses. Consequently, “general virucidal activity” can be claimed. Moreover, we have extended this synergistic action to bacteria (*P. aeruginosa*, EN 13727). Based on physicochemical investigations, we have proposed two independent mechanisms of action against bacteria and non-enveloped viruses, operating at sub- and super-micellar concentrations, respectively. This synergistic mixture could then be highly helpful as a universal disinfectant to avoid the spread of infectious viral or bacterial diseases in community settings, including COVID-19 and monkeypox (caused by enveloped viruses).

## 1. Introduction

Since 2019, a human coronavirus, SARS-CoV-2, has emerged, leading to a pandemic named COVID-19 [1]. This disease is principally transmitted from host-to-host after close contact through respiratory droplets and aerosols produced when an infected person coughs, sneezes, or talks, rather than by touching an infected surface or object (also referred to as fomites) [2]. In May 2022, a new outbreak caused by the monkeypox virus (MPXV) was reported [3]. Based on current data, the major routes of MPXV transmission are close contact with skin or genital lesions of infected people or indirect contact with objects contaminated by bodily fluids [3]. Surprisingly, a large number of infections have been reported in men who have sex with men due to the coincidental introduction of MPXV to this community and its spread through intimate contact during sexual activities [3]. However, MPXV was recently detected in sperm and pre-ejaculate, highlighting the possibility of sexual transmission and making some populations more vulnerable [4]. Gays, bisexuals, and male sex workers performing unprotected oral and anal intercourse with a high turnover of partners are particularly exposed when pre-ejaculate or semen is expelled after sexual arousal or ejaculation. Nevertheless, this spread would not limit to same-sex intercourse; any sexual contact involving mucosa could be also a high-risk activity. A notable feature of SARS-CoV-2 and MPXV is that these infectious agents are enveloped viruses, meaning that the nucleic acid is surrounded by a protein capsid and an additional lipid outer bilayer [3,5]. These kinds of viruses have “limited” survival outside host environments and are easily inactivated by various physical (e.g., heat, light) and chemical agents (e.g., detergents) [6]. In contrast, non-enveloped viruses (e.g., poliovirus and norovirus) show much higher resistance [7]. Consequently, enveloped viruses are more susceptible to chemical agents than non-enveloped viruses [8]. By way of example, detergents can easily inactivate enveloped viruses by disrupting the lipid envelope but are inactive against non-enveloped ones, which require the dislocation of the capsid [9].

**Figure 1:**
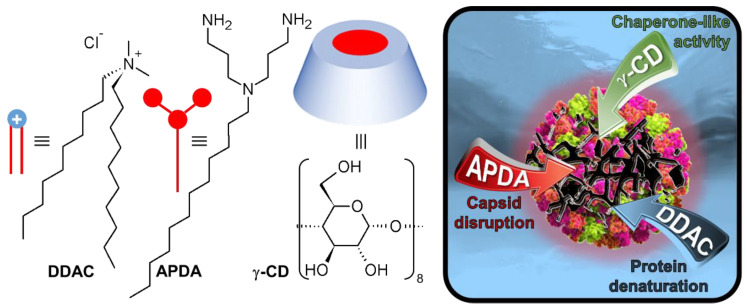
Structure, representation, and effects on non-enveloped viruses of didecyldimethylammonium chloride (**DDAC**), *N*,*N*-bis(3-aminopropyl)dodecylamine (**APDA**) and γ-cyclodextrin (**γ-CD**).

To reduce virus transmission by fomite route, the disinfection of surfaces must be used as a prevention measure. Although surfaces seem to play a minor role in the transmission of SARS-CoV-2, poliovirus shows a much higher persistence on indoor surfaces where they may remain infective for weeks [10]. To demonstrate the effectiveness of disinfectant formulations, standard methods are used. According to the EN 14476 standard, a formulation must demonstrate virucidal efficacy against non-enveloped viruses to claim “*general virucidal activity*”, which means that this disinfectant is effective against these viruses and also against all others not tested, including enveloped viruses, and, in particular, SARS-CoV-2 or MPXV [11]. In healthcare systems, *N*,*N*-didecyl-*N*,*N*-dimethylammonium chloride, **DDAC**, and/or *N*,*N*-bis(3-aminopropyl)dodecylamine, **APDA**, are widely used to prevent and control infections by the reduction of germs on hard surfaces (Figure 1) [12]. These compounds have broad-spectrum activity against bacteria and fungi. However, the susceptibility of viruses to **DDAC** is limited to the presence of a viral envelope, as it is a membrane disruptor [12]. On the other hand, native cyclodextrins (**CDs**) are known to boost the virucidal action of **DDAC** against enveloped viruses, since both of them interact with lipids [13]. Unfortunately, native **CDs** alone or in a mixture with **DDAC** have no activity against non-enveloped viruses [13]. In contrast, **APDA** can be active against non-enveloped viruses but at high concentrations. To our knowledge, its mechanism remains unexplored. However, as **APDA** is an *N*-alkylated norspermidine and as viruses use polyamines in numerous stages of their life cycle (e.g., the packing of RNA or DNA by charge neutralization [14]), we can logically propose the following mechanism for **APDA**: (*i*) the protonation of the amino groups occurs, (*ii*) the viral proteins are impacted by pH changes facilitating the passage of the positively charged **APDA** through the capsid, (*iii*) the **APDA** cations screen some negative charges of the viral nucleic acid modifying the hydrophilic/lipophilic balance, and (*iv*) the capsid is disrupted.

Based on these considerations, it may be hypothesized that synergistic effects can be obtained by combining **APDA**, **DDAC,** and **γ-CD** against non-enveloped viruses. Indeed, **APDA** is able to weaken the capsid integrity, whereas **APDA** and **DDAC** can participate in the capsid dissociation, and **γ-CD** can be used to prevent the re-aggregation of proteins by chaperone-like activity [15]. In this paper, as it is much more challenging to inactivate non-enveloped viruses, we have chosen poliovirus and norovirus as non-enveloped virus models in order to develop a universal disinfectant because, with these two viruses, we can extrapolate a “*general virucidal activity*”. Finally, the scope of this system has been extended to *P. aeruginosa*, the most commonly isolated nosocomial bacterial pathogen.

## 2. Materials and Methods

### 2.1. General Information

Didecyldimethylammonium chloride (**DDAC**) was synthesized according to the procedure described in our previous work [12]. *N*,*N*-Bis(3-aminopropyl)dodecylamine (**APDA**) was purchased from TRC (Toronto, ON, Canada). The other chemicals were obtained from Merck at the highest purity available (>99.9%). All experiments were assayed in triplicate with solutions prepared extemporaneously with sterile water purchased from Fischer Scientific SAS (Illkirch-Graffenstaden, France). The neutralizer and diluent composition was Tween 80 (30 mL), phosphatidylcholine (3 g), sodium thiosulfate pentahydrate (5 g), histidine chlorhydrate (1 g), tryptone salt (9.5 g), saponin (30 g), water (qs. 1 L). pH was measured with a pH330i (WTW, Bremen, Germany).

### 2.2. Surface Tension Measurements

Equilibrium surface tension measurements were obtained using K100MK2 tensiometer (Krüss, Hamburg, Germany) equipped with a platinum plate. The measurement was carried out in a circular thermostated dish maintained at 25 ± 0.05 °C using a circulating water bath. Before each experiment, the plate was flame cleaned. All surface tension values were averaged over at least three measurements (S.D. ± 1.5% of the mean).

### 2.3. Iterative Algorithm for Fitting Treatment

The theoretical surface tension data (σ_cal_) may be calculated with the suitable equations to fit the constant (K_ads_ or K_ass_, see below) with a homemade algorithm [16]. The best fit was obtained by choosing the constant that minimizes the distance between the theoretical function (σ_cal_) and the experimental data set (σ_obs_). The standard deviation (SD) was calculated for two values K and K′ (K′ = K − I with I an increment) as well as the coefficient of determination, R^2^:

(1) $$R^{2}=1-\sum^{\;}{\left(\sigma_{\mathrm{obs}}-\sigma_{\mathrm{cal}}\right)}^{2}/\sum^{\;}{\left(\sigma_{\mathrm{obs}}-\bar{\sigma_{\mathrm{obs}}}\right)}^{2}$$

The best-fit value is obtained for minimum ΔSD:

(2) $$\Delta \mathrm{SD}=\left|{\mathrm{SD}}_{K}-{\mathrm{SD}}_{K’}\right|$$

### 2.4. Virucidal Assay

All virucidal tests were carried out in accordance with EN 14476 standard using poliovirus type 1 (LSc-2ab from Eurovir, propagated on HeLa cells in Dulbecco’s modified Eagle, DMEM, medium supplemented with 10% fetal bovine serum) and murine norovirus (S99 Berlin from Friedrich-Loeffler-Institut, propagated on RAW 264.7 in DMEM with 1 g/L glucose). Cells were infected with virus at a Multiplicity of Infection (MOI) of 0.1-1 for 2~3 days. The cellular debris of the stock virus suspension was separated by low-speed centrifugation. The virus titers of these suspensions ranged from 10^8^ to 10^9^ TCID_50_/mL (tissue culture infectious dose 50). Another sample stock solution of **DDAC**, **APDA**, and/or **γ-CD** (for final compositions, see below) was prepared. Furthermore, 200 µL of the sample (as delivered or diluted with distilled water) was added to 200 μL of a viral suspension supplemented by bovine serum albumin (3 g/L). After incubation for 30 min ± 10 s (or 60 min ± 10 s) at 20 ± 1 °C, the mixtures were neutralized by dilution or molecular sieving using MicroSpin^TM^ S 400 HR (Sigma-Aldrich, Burlington, MA, USA) (for formaldehyde only, positive control). A sample (100 µL) for each dilution was used to infect four replicate wells in 96-well microtiter plates (Nunclon™ Delta Surface, Thermo Scientific™ Nunc™, Thermo Fisher Scientific, Waltham, MA, USA) containing 100 μL cell suspension. After 7 days of inoculation at 36 °C ± 1°C, cultures were observed for cytopathic effects. The virus titers were determined using the Spearman and Kaerber method. The virucidal activity was defined as the difference between the log titer of the control minus the log titer of the test solution [17,18]. This difference is presented as a reduction factor with a 95% confidence interval. According to EN 14476, the minimum virucidal concentration (MVC) is defined as the lowest concentration, giving at least 4-log_10_ (99.99%) reduction. Note that the virus titers at the beginning and at the maximum contact time without disinfectant were used as negative controls whereas the positive control was achieved using 0.7 wt.% formaldehyde after 30 and 60 min (for additional details on controls, see the EN 14476 standard) [11].

### 2.5. Bactericidal Assay

Bactericidal tests were carried out in accordance with European standard EN 13727 using *Pseudomonas aeruginosa* (ATCC^®^ 15442™). The prepared solution (see below) was diluted with sterile water and a suspension of *P. aeruginosa* was added (with bovine serum albumin, 3 g/L). The bacteria titer was adjusted between 1.5 × 10^7^ and 5.0 × 10^7^ colony-forming unit per millimeter (CFU/mL). The sample was maintained at 20 ± 1 °C for 5 min ± 10 s. To determine the efficacy of the antimicrobial solution, an aliquot was taken. The bactericidal activity was neutralized immediately by dilution (see above) to prevent an overestimation of efficacy. Samples were serially diluted from 10^−1^ to 10^−5^ and each dilution was plated in duplicate on tryptone soya agar. After 48 h incubation at 37 ± 1 °C, the number of viable bacteria in each sample was estimated by dividing the number of colonies by the dilution factor (CFU/mL). This difference is presented as a reduction factor with a 95% confidence interval. According to the EN 13727 standard, a reduction factor ≥5-log_10_ is considered evidence of bactericidal activity. The minimum bactericidal concentration (MBC) is defined as the lowest concentration giving at least 5-log_10_ (99.999%) reduction. Note that the bacteria titers at the beginning and at the maximum contact time without disinfectant were used as negative controls whereas the positive control was achieved using 30 μg/mL of cetyl trimethyl ammonium bromide (for more details, see the EN 13727 standard).

## 3. Results and Discussion

### 3.1. Theoretical Considerations

**Figure 2:**
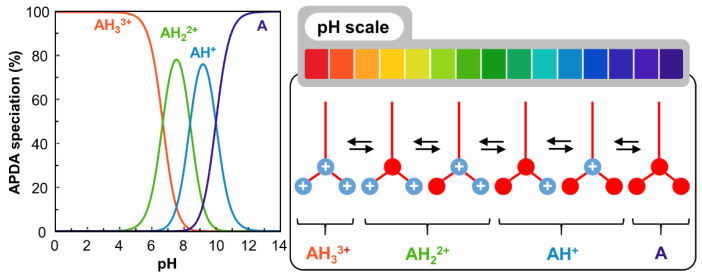
Protonation and charge states of **APDA** as a function of pH.

From a physicochemical point of view, **APDA** is an amphiphilic molecule bearing both a hydrophobic part and a hydrophilic part. The hydrophobic part is composed of a 12-carbon aliphatic chain, while the hydrophilic part contains one tertiary and two ionizable primary amines. Therefore, three pKa are reported in the literature: pKa_1_: 6.7; pKa_2_: 8.4; and pKa_3_: 10.0 [19]. pKa_1_ is attributed to the tertiary amine, while pKa_2_ and pKa_3_ are attributed to the primary amines [19]. Consequently, depending on the pH, **APDA** will occur in different forms noted A for the non-protonated one to AH_3_^3+^ for the most protonated one (Figure 2). These different structural states will give the molecule tunable surfactant properties as a function of the pH: at pH values greater than 10 (alkaline medium), non-protonated **APDA** has a hydrophobic character, whereas at pH values below 6 (acidic medium), **APDA** is well protonated and hydrophilic.

**Figure 3:**
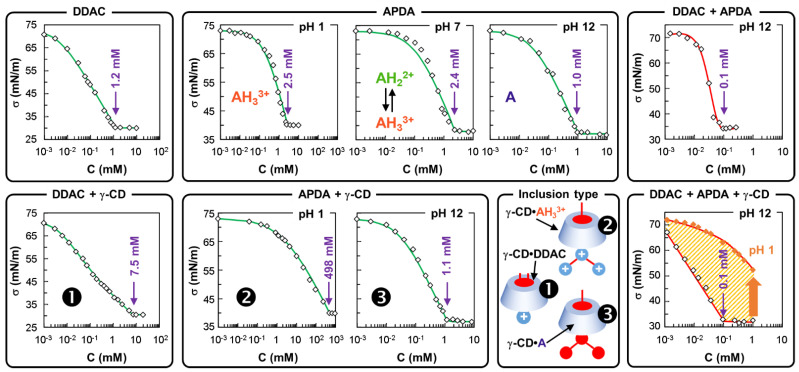
Surface tension (σ) plotted against surfactant concentration at 25.0 °C with or without **γ-CD** (*NB.* all binary or ternary systems are equimolar). The green lines represent the modeling and are calculated from Equations (3), (4), (7) and (8) (see below for the best fit parameter values). The red lines are not modeled. For **DDAC**/**APDA**/**γ-CD** ternary mixtures, the effect of acidification of the surface tension is also shown in orange.

Next, the surface tension isotherms of **DDAC** and **APDA** (at pH 1, 7 and 12) without **γ-CD** are shown in Figure 3.

As depicted in Figure 3, the **γ-CD** alone is not surface active at air/water interfaces [20]. In contrast, the surface tension isotherms of **DDAC** and **APDA** are typical of surfactants (Figure 3). In fact, the surface tension of water decreases by adding surfactant until it reaches a minimum value (σ^∞^). The concentration of surfactant where that minimum is attained is usually described as the CMC. Indeed, at this point, extra surfactant ends up as micelles in the water rather than doing any further reduction in surface tension. The CMC value is 1.2 mM for **DDAC** whatever the pH. Obviously, the CMC values for **APDA** substantially depend on the degree of neutralization of the amine functions: 2.5, 2.4, and 1.0 mM at pH 1, 7, and 12, respectively (see Figure 3 and Table 1). Indeed, protonated amines form micelles less easily (2.5 mM at pH 1), while unprotonated amines exhibit easier micellization (1.0 mM at pH 12). In other words, the micellization is hindered by the electrostatic repulsion between the positively charged ammonium heads. The CMC as well as σ^∞^ have been reported in Table 1.

The surface tension isotherms give us another set of values, Γ_max_ (the limiting surface concentration) and K_ads_ (the absorption constant) which are of great importance. In each case, the experimental data sets can be fitted with the Langmuir-Szyszkowski isotherm (Equation (3)). Indeed, at a surfactant concentration of C, the surface tension σ depends on the surface tension of the solvent σ_0_ (72.8 mN/m at 25 °C for water) and on the two constants K_ads_ and Γ_max_:

(3) $${\sigma =\sigma }_{0}-{\mathrm{nRT}\Gamma }_{\max}\ln\left({1+K}_{\mathrm{ads}}C_{S}\right)$$

where R is the ideal gas constant, T is the temperature, C_S_ is the surfactant concentration, and n is the dissociation parameter. It is noteworthy that the Γ_max_ is obtained from the linear regression analysis of the σ versus lnC straight lines at a value just below the CMC using the Gibbs isotherm (Equation (4)).

(4) $$\Gamma =-1/\mathrm{nRT}{\left(d\sigma /{\mathrm{dlnC}}_{S}\right)}_{P,T}$$

In the present work, we considered that n = 1 for all investigated surfactants. In such a condition, there is a clear impact on the value of Γ_max_ but not on the relevant value of K_ads_. The K_ads_ value is estimated at 2.41 × 10^5^ M^−1^ for **DDAC** (see Table 2). Obviously, the K_ads_ values for **APDA** depend on the degree of neutralization of the amine functions: 4.5 × 10^3^, 8.0 × 10^3^ and 2.8 × 10^4^ M^−1^ at pH 1, 7 and 12, respectively (see Table 2). As K_ads_ reflects the preference for the surfactant to be at the interface rather than in the water, the higher K, the lower the CMC, because the surface will reach saturation faster. Consequently, explanations similar to those given for CMCs can be made (see above).

To get better insights into the influence of **γ-CD**, we recorded the surface tension of **DDAC** or **APDA** with **γ-CD** (Figure 3). The surface activity of **DDAC** or **APDA** at pH 1 is clearly modified with the addition of **γ-CD** whereas no clear variation is observed with **APDA** at pH 12. As aqueous solutions of **γ-CD** do not have any surface activity, this demonstrates that **γ-CD** forms inclusion complexes with **DDAC** and protonated **APDA** whereas, with non-protonated **APDA**, the association constant is very weak. On the other hand, the surface tension values of **DDAC** or **APDA** above the CMC remain the same as that of **DDAC** or **APDA** alone (see Table 1). This observation indicates that the inclusion complexes have no surface activity (as the **γ-CD**) and that there is little interaction between the complexes and the **DDAC** or **APDA** micelles [16]. Therefore, the combination of the Szyzkowski equation and the mass balance equations for each component can be used to determine the association constant (K_ass_) between single surfactant (S: **DDAC** or **APDA**) and **γ-CD**. If we assume that only the 1:1 inclusion complex is formed, the mass balance equation can be expressed as:

(5) $$C_{\mathrm{CD}}=\left[\mathrm{CD}\right]+K_{\mathrm{ass}}\left[S\right]\left[\mathrm{CD}\right]$$

(6) $$C_{S}=\left[S\right]{+K}_{\mathrm{ass}}\left[S\right]\left[\mathrm{CD}\right]$$

where C_CD_ is the total **CD** concentration, [S] is the uncomplexed monomer of S (able to adsorb at the air/water interface), and [CD] is the free **CD** concentration. From Equations (3) and (4), [CD] and [S] can be calculated as follows:

(7) $$\left[\mathrm{CD}\right]+\left(K_{\mathrm{ass}}C_{S}\left[\mathrm{CD}\right]\right)/\left(1+K_{\mathrm{ass}}\left[\mathrm{CD}\right]\right)-C_{\mathrm{CD}}=0$$

(8) $$\left[S\right]{=C}_{S}/\left({1+K}_{\mathrm{ass}}\left[\mathrm{CD}\right]\right)$$

The association constant K_ass_ can be estimated with an appropriate algorithm from the experimental surface tension isotherm in the presence of **CD** using Equations (7), (8), and (3). The green lines in Figure 3 are the best fits, assuming the prevalence of a 1:1 complex for all **DDAC/γ-CD** and **APDA/γ-CD** mixtures. As depicted in Table 2, the 1:1 binding constants with **γ**-**CD** are in the order: **APDA** (pH 1) **>> DDAC** > **APDA** (pH 12).

**Figure 4:**
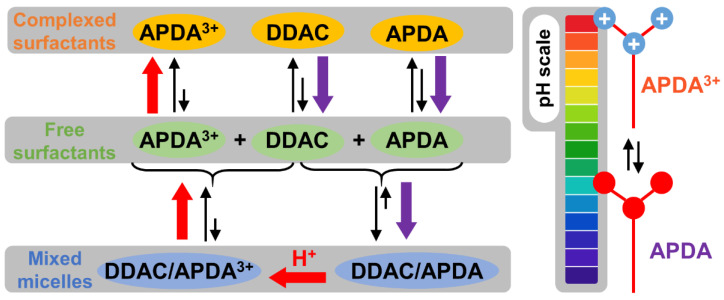
Equilibria between free, complexed, or co-micellized surfactants as a function of pH. The arrows show the equilibrium shift in acidic (red) and basic medium (purple).

It is noteworthy that the equilibria between free, complexed, or co-micellized surfactants as a function of pH is summarized in Figure 4 in order to facilitate the following discussion.

Next, we have investigated the surface tension of **DDAC/APDA** mixtures in equimolar conditions at pH 12 (Figure 3). It is clearly shown a nonideal behavior between the two surfactants. Indeed, according to Clint, the CMC of an ideal surfactant binary mixture is given by the following equation:

(9) $$1/\mathrm{CMC}=\alpha_{1}/C_{1}+\alpha_{2}/C_{2}$$

where α_i_ and C_i_ are the stoichiometric mole fraction and the CMC in the mixed aggregate of the ith component in the mixture (i = 1 for **DDAC** and 2 for **APDA**). Therefore, the experimental CMC of the mixture (0.1 mM) is much lower than the calculated CMC considering an ideal behavior (1.09 mM). This non-ideal behavior is attributed to a synergistic effect resulting from an attractive interaction between the two surfactants, leading to a stabilization of the micellar phase. In other words, the synergism in the aggregated system indicates that the strength of the attractive interaction between **DDAC** and **APDA** surfactants is stronger than the self-attraction of individual surfactants. In the present case, interactions through electrostatic forces between the quaternary ammonium group of **DDAC** and the nitrogen of the amines can be expected. Indeed, the repulsive interactions between the ionic head groups of **DDAC** can be counterbalanced by the **APDA** in order to decrease the electrostatic repulsion between the cationic groups, leading to a stabilization of the mixed micelles. In the presence of **γ-CD**, the CMC remains unmodified (0.1 mM), but the adsorption process is clearly modified because the slope of the surface tension isotherm in the premicellar region is affected (see Figure 3). This observation suggests that the two mixed surfactants do not adsorb synchronously in the presence of **γ-CD**. Indeed, as the **γ-CD** has more affinity for **DDAC** than **APDA** at pH 12 (see K_ass_ in Table 2), **APDA** and some free **DDAC** adsorb first at the air/water interface. Furthermore, **DDAC** is progressively dissociated to allow its adsorption, which tends towards a final composition similar to the mixed monolayer obtained without **γ-CD** as the total concentration increases. In other words, the complexes act as a reservoir of **DDAC** molecules, which are readily available for adsorption: the **γ-CD** delays the **DDAC** adsorption. Therefore, the strong synergistic effect resulting from attractive interactions between ammonium cation and amine residues is stronger than the **DDAC/γ-CD** complexation. Obviously, these strong interactions can be easily overcome by acidification (see Figure 3). Indeed, the acidification results in highly charged mixed micelles leading to electrostatic repulsions between the charged ammonium groups and facilitating the complexation of **APDA** and, to a lesser extent, of **DDAC** (see K_ass_ in Table 2). However, it is assumed that the mixed micelles are not totally dissociated, as the hydrophobic interactions between alkyl tails of the two surfactants also contribute to the formation of mixed aggregates.

It is noteworthy that we have chosen to work with a reduced concentration of **γ-CD** to avoid the formation of the complex. Consequently, in the following discussion, the general composition of the **DDAC**/**APDA**/**γ-CD** ternary mixture (i.e., the sample stock solution) is 4 mM of **γ-CD** supplemented with 33 mM of **DDAC** and 33 mM of **APDA**.

### 3.2. Virucidal Performance against Poliovirus

**Figure 5:**
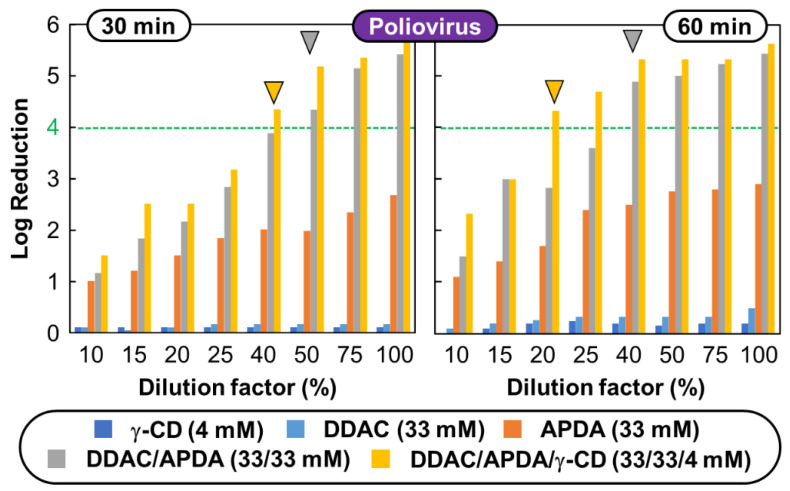
Virucidal activity in log_10_ titer reduction factor as a function of dilution factor recorded at room temperature against poliovirus type 1 (LSc-2ab). Minimum virucidal concentration (MVC) is pointed on the graph by a triangle (if applicable). The standard deviation on the values is ±6%. The initial pH of all **APDA** solutions were around ~10.5.

Virucides that inactivate viral particles outside the cell (virions) by damaging their envelopes, capsids, or genomes are widely used to disinfect hard surfaces, surgical instruments, etc. This practice is useful for the prevention of viral illnesses in community settings and households [12,13]. As previously mentioned, non-enveloped viruses are more resistant to chemical agents [7,9,10]. However, among these viruses, Poliovirus is much more resistant than the others: it is used as a reference virus in the European standard EN 14476 (see above). Therefore, we first determined the virucidal activity of the **DDAC**/**APDA**/**γ-CD** ternary system against poliovirus type 1 (PV-1). In control experiments, we evaluated the virucidal activity of each compound alone or in binary mixture against PV-1 at various concentrations. The dose-dependent virucidal activity against PV-1 is presented in Figure 5.

Before analyzing the results shown in Figure 5, five control experiments were made to prove the viability of the obtained data according to EN 14476 standard. The five validation tests are: (A) negative control (NC), which determines the infectivity of the virus suspension at the beginning and at the maximum contact time without disinfectant, (B) cytotoxicity effect (CE) to ensure that cells are not altered, (C) suppression efficiency (SE), which verifies that the neutralizing method is efficient to suppress the virucidal activity, (D) interference control (IC), which verifies the susceptibility of infection in cells is not influenced negatively by the disinfectant (this value is compared with phosphate-buffered saline solution, (IC_PBS_), and (E) positive control (PC), which ensures the virus can be inactivated by a classical antimicrobial agent (i.e., formaldehyde) after 30 and 60 min. By way of example, the controls tests and method validation results, expressed as log_10_ TCID_50_ per mL, for PV-1 and/or HeLa cells and/or disinfectant at dilution factors of 100% (33 mM of **DDAC**, 33 mM of **APDA** and 4 mM of **γ-CD**) when appropriated, are NC = 7.00 ± 0.38, CE = 1.50 ± 0.00, SE = 5.88 ± 0.37, IC = 7.25 ± 0.33 (IC_PBS_ = 7.50 ± 0.00) and PC = 1.00 and 3.00 ± 0.00 after 30 and 60 min. As these results proved the viability of the EN 14476 method used, the virucidal activities reviewed in the following discussion are only due the ability of the disinfectant to produce a reduction in the number of viable PV-1.

It is noteworthy that a reduction in virus titer of ≥4-log_10_ (equivalent to a ≥99.99% reduction of virus titer) was required to claim a virucidal, disinfectant and antiseptic efficacy according to EN 14476 [11]. Therefore, the minimum virucidal concentration (MVC) is determined as the lowest concentration that results in at least a 99.99% reduction of the original virus titer. As depicted in Figure 5, all individual components (**DDAC** or **APDA** or **γ-CD)** did not pass the test according to the NF 14476 standard against PV-1: the reduction of virus titer of <4-log_10_ after 30 or 60 min of contact time. However, it is noteworthy that **APDA** shows a weak but higher virucidal activity (a reduction in virus titer of ~3-log_10_ after 60 min) compared with **DDAC**. In contrast, **DDAC**/**APDA** and **DDAC**/**APDA**/**γ-CD** mixtures demonstrate virucidal efficacy against PV-1 (i.e., ≥4-log_10_ reduction). Indeed, after 30 min of contact time, the MVC were obtained at dilution factors of 50% (16.5 mM of **DDAC** and 16.5 mM of **APDA**) and 40% (13.2 mM of **DDAC**, 13.2 mM of **APDA** and 1.6 mM of **γ-CD**) for **DDAC**/**APDA** and **DDAC**/**APDA**/**γ-CD** mixtures, respectively. This observation confirms a clear synergistic effect for the two mixtures due to the concomitant effects of each compound on the viral integrity. Obviously, this effect was more marked after 60 min: ≥4-log_10_ reduction was observed at dilution factors of 40% (13.2 mM of **DDAC** and 13.2 mM of **APDA**) and 20% (6.6 mM of **DDAC**, 6.6 mM of **APDA** and 0.8 mM of **γ-CD**) for **DDAC**/**APDA** and **DDAC**/**APDA**/**γ-CD** mixtures, respectively. For both mixtures, it is noteworthy that the MVC values are higher than the CMC of the respective mixtures (0.1 mM; see Table 1). All these observations support that: (*i*) free **DDAC** and **γ-CD** are unable to inactivate PV-1 at concentrations ≤33 and ≤4 mM, respectively, after 30 or 60 min of contact time, (*ii*) free **APDA** exhibits weak virucidal activity, (*iii*) the **DDAC**/**APDA** mixture shows a synergistic effect in term of micellization (see earlier) and viral inactivation, and (*iv*) the **DDAC**/**APDA**/**γ-CD** mixture does not show an effect on micellization (see above) but an additional effect against PV-1. All these facts suggest that the **APDA** molecules are the primary active species. It is noteworthy that the initial pH of all investigated **APDA** solutions was around ~10.5. Therefore, the **APDA** triamines, located in mixed **DDAC**/**APDA** micelles, can easily be protonateds which induces the following steps: *(i)* the mixed micelles become highly charged leading to electrostatic repulsions, facilitating the mixed micelles dissociation, (*ii*) the viral capsid proteins are impacted by pH changes facilitating the passage of more and more **APDA** or **DDAC** through the capsid, (*iii*) the cations screen the negative charges of the viral nucleic acid, (*iv*) the capsid is fully dislocated, and (*v*) the **γ-CDs** prevent the re-aggregation of proteins via chaperone-like activity [15]. It is noteworthy that co-micelles as well as the various inclusion complexes can be seen as reservoirs of **APDA** and **DDAC**, which are readily available for interaction with the viral capsid.

### 3.3. Virucidal and Biocidal Performance against Norovirus and Pseudomonas aeruginosa

**Figure 6:**
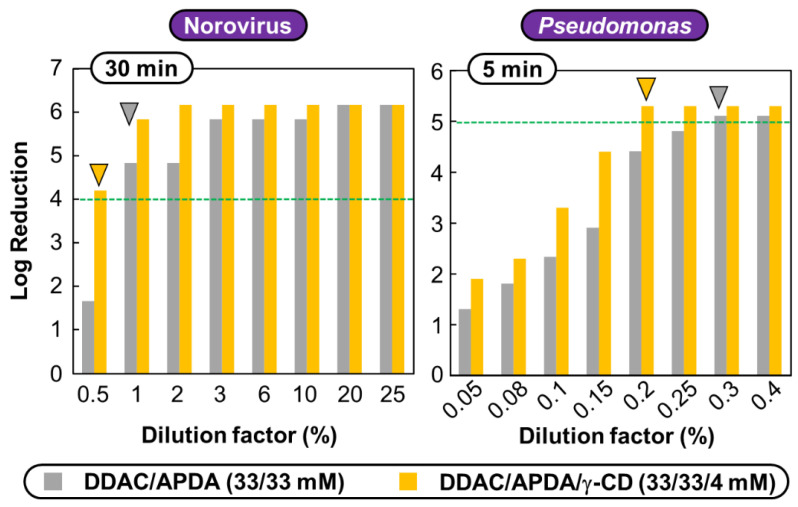
Virucidal or biocidal activity in log_10_ titer reduction factor as a function of dilution factor recorded at room temperature against non-enveloped virus (murine norovirus, S99 Berlin) and bacterium (*P. aeruginosa*, ATCC^®^15442^TM^). The standard deviation on the values is ±6%. Minimum virucidal concentration (MVC) or minimum bactericidal concentration (MBC) is pointed on the graph by a triangle. The initial pH of all **APDA** solutions were around ~10.5.

To extend the scope of the systems based on **DDAC**, **ADPA**, and **γ-CD**, the biocidal action against non-enveloped viruses and bacteria was carried out under similar conditions on the well-known murine norovirus (MNV) and *Pseudomonas aeruginosa*. The MNV was chosen because it is also a model used in EN 14476 standard (see above). Moreover, MNV affects mice, and it is commonly used in research to model Human norovirus [21]. In contrast, *Pseudomonas aeruginosa* (gram-negative bacterium) can be highly pathogenic for humans under certain conditions. Indeed, serious infection often occurs during existing diseases or conditions (e.g., cystic fibrosis and traumatic burns). The treatment of *P. aeruginosa* infections can be difficult due to its natural resistance to antibiotics, as it becomes more and more often responsible for nosocomial infections [22]. The mortality rate reaches up to 61% in immunocompromised patients [23]. To prevent this nosocomial infection, hospitals’ sanitation protocols include hard surface disinfection. Consequently, **DDAC**/**APDA** and **DDAC**/**APDA**/**γ-CD** mixtures have been tested against these two pathogens (Figure 6). The controls tests and method validation results, expressed as log_10_ TCID_50_ per mL, for MNV and/or RAW 264.7 cells and/or disinfectant at dilution factors of 25% (8.25 mM of **DDAC**, 8.25 mM of **APDA** and 1 mM of **γ-CD**) when appropriated, are: NC = 6.63 ± 0.25, CE = 1.50 ± 0.00, SE = 6.75 ± 0.33, IC = 7.00 ± 0.38 (IC_PBS_ = 6.75 ± 0.33) and PC = 6.00 ± 0.00 after 30 and 60 min. Consequently, the recorder virucidal activities are only due to the ability of the disinfectant to produce a reduction in the number of viable MNV. For *P. aeruginosa*, the bacteria titers at the beginning and at the maximum contact time without disinfectant were used as overall negative controls. On the other hand, a positive-control was achieved using 30 μg/mL of cetyl trimethyl ammonium bromide.

As expected, the two mixtures are highly effective against MNV because this last is more sensitive than PV-1 (see above). Indeed, after 30 min, a reduction in virus titer of ≥4-log_10_ (corresponding to an inactivation of ≥99.99%) was obtained at dilution factors of 1% (0.33 mM of **DDAC** and 0.33 mM of **APDA**) and 0.5% (0.165 mM of **DDAC**, 0.165 mM of **APDA** and 0.02 mM of **γ-CD**) for **DDAC**/**APDA** and **DDAC**/**APDA**/**γ-CD** mixtures, respectively. For both mixtures, it is noteworthy that the MVC values are higher than the CMC of the respective mixtures (0.1 mM; see Table 1). These observations confirm the previously reported synergistic effect for the two mixtures due to the concomitant effects of each compound on the viral integrity (for the mechanism of action; see above). In comparison, the MVC values obtained for DDAC/γ-CD (equimolar condition) against herpes simplex type 1, respiratory syncytial and vaccinia virus (i.e., enveloped viruses) were 35, 75 and 75 µM (each compound), respectively [24]. In contrast, coxsackievirus B4 (non-enveloped virus) was not inactivated by **DDAC** and/or **γ-CD** as the virus susceptibility to chemical biocides is in the following order: enveloped >>> non-enveloped [25,26].

Similar effects were observed against *P. aeruginosa*. Indeed, after 5 min, a reduction in virus titer of ≥5-log_10_ (corresponding to an inactivation of ≥99.999%) was obtained at dilution factors of 0.3% (0.099 mM of **DDAC** and 0.099 mM of **APDA**) and 0.2% (0.066 mM of **DDAC**, 0.066 mM of **APDA** and 0.008 mM of **γ-CD**) for **DDAC**/**APDA** and **DDAC**/**APDA**/**γ-CD** mixtures, respectively. This differential susceptibility can first be correlated with the well-known disinfection scale: the pathogen susceptibility to chemical biocides is in the following order: enveloped viruses >> bacteria >>> non-enveloped viruses [25]. However, a careful look at the physicochemical data reveals that, for both mixtures, the MBC values are lower than the CMC of the respective mixtures (0.1 mM). This observation supports that a change in the mechanism of action takes place between non-enveloped viruses and bacteria. Indeed, gram-negative bacteria have an inner cell membrane (cytoplasmic) and an outer membrane containing lipopolysaccharides and phospholipids [12]. Based on this, we can reasonably suppose that the adsorption of **DDAC** and **APDA** cations leads to their insertion in the outer membrane associated with a rapid flip-flop across the lipid membrane leading to the alteration of the membrane. Consequently, the outer membrane of gram-negative bacteria is disrupted, leading to a loss of cell viability. In the presence of **γ-CDs**, this alteration facilitates the phospholipids extraction by complexation [24,26,27]. Similar mechanisms have already been reported for **DDAC** and **DDAC**/**γ-CD** on enveloped viruses [12,13,24,26,27]. Therefore, if high biocide concentrations are required to inactivate non-enveloped viruses by protein denaturation, the phospholipid perturbation is easily achieved at sub-micellar concentration for gram-negative bacteria. It is noteworthy that pH change can also be invoked in the mechanism of action as well as interference with cytoplasmic proteins and nucleic acids for high disinfectant concentrations. All these effects act concomitantly on bacterial integrity.

### 3.4. Comparison with other Disinfection Systems

It should be noted that the quaternary ammonium surfactants are currently widely used in the vast majority of consumer and industrial formulations on the market for their biocidal properties against bacteria and enveloped viruses by disrupting their phospholipid bilayers [28]. For instance, household disinfectants classically contain benzalkonium, didecyldimethyl ammonium, alkyl dimethyl benzyl ammonium, cetyl pyridinium, dioctyldimethyl ammonium, benzethonium and/or mecetronium cations typically balanced by chloride, bromide and/or sulfate anions [29]. This class of cationic surfactants are largely used because their biocidal activity is maintained in hard water and in the presence of anionic and/or organic residues [30]. Moreover, these cationic salts are stable, odorless, colorless, and relatively nontoxic [31]. As their biocidal activity depends on concentration, duration of application and temperature, the opportunity for direct comparisons between different studies or formulations is extremely reduced. However, it is admitted that cationic surfactants alone, even those with high bactericidal effects, are ineffective against non-enveloped viruses [32]. By way of example, Laurent and coworkers reported the inactivation effect of benzalkonium chloride on enveloped viruses (e.g., herpes simplex virus, HSV, cytomegalovirus, CMV, and respiratory syncytial virus, RSV) and non-enveloped viruses such as adenovirus, ADV, enterovirus, ENV, and BK virus, BKV [33]. The authors highlighted that the in vitro sensitivity to benzalkonium cation (after 60 min of exposure at 0.141 µM) was high against enveloped viruses (reduction in virus titer of >3-log_10_) but lower against non-enveloped viruses (reduction in virus titer of <3-log_10_). Another typical example is given by Shirai et al. for the didecyldimethyl ammonium cation [34]. Indeed, **DDAC** was very effective against the African swine fever virus (enveloped), but it was less effective against swine vesicular disease virus (non-enveloped), even to a tenfold higher working concentration than one used with the enveloped virus [35]. Electron microscopic observation revealed that the cationic surfactant induced disruption of the lipid outer membrane of the enveloped viruses as previously mentioned. Fortunately, **DDAC** became effective against the non-enveloped virus with an increase in pH of the solution by the addition of 0.05% NaOH [34]. Consequently, in order to boost inactivation, ammonium-based biocides are usually used in a mixture with other biocidal compounds such as other cationic surfactants, alcohols (e.g., isopropanol, ethanol), organic acids (e.g., citric acid) and/or formaldehyde [26,36,37]. The modification of the anion can also be used to enhance biocidal activities [38]. Specifically, Zonta and coworkers proved that **DDAC** in combination with glutaraldehyde and isopropanol enhance the reduction of infectivity of non-enveloped viruses (e.g., feline calicivirus, FCV) [39]. As native **CDs** are known to enhance antibacterial and antifungal activity, some studies described their use in combination with ammonium surfactants [12,13,24,27]. However, the effect of these solutions is limited to enveloped viruses, even when mixed with other biocides (e.g., ethoxylated nonionic surfactants) [26]. From a more general point of view, Health Canada reports that for 969 approved or marketed disinfectants, only 61 products use an ammonium salt alone and 3 are up to 6 active ingredients [40]. Among the ready-to-use disinfectant solutions for hard surfaces which use only one active ingredient, the lowest DDAC concentration is obtained for the “Lemon Drop”, marketed by Ostrem Chemical Co Ltd. (0.045 wt.% or 1.24 mM) [41]. This commercial disinfectant, used in institutions, hospitals and food processing areas, is effective against bacteria (e.g., *P. aeruginosa*, ATCC^®^ 15442^TM^ and *Staphylococcus aureus*, ATCC^®^ 6538^TM^) and enveloped viruses (e.g., SARS-CoV-2) [41]. However, our new formulation is more effective than the commercial one because at least 99.999% (Δlog_10_ = 5) of *P. aeruginosa* are killed at a total active ingredient concentration of 0.14 mM (0.066 mM of **DDAC**, 0.066 mM of **APDA** and 0.008 mM of **γ-CD**) in 5 min of contact whereas only 99.9% (Δlog_10_ = 3) is observed at 1.24 mM after 10 min for the commercial product [41]. The improvement of biocidal activity combined with the noticeable reductions of the contact time (by a factor of 2), the total active ingredient concentration (by a factor of 8.9) and the partial replacement of hazardous biocides by the nontoxic **γ-CD** is of great interest in the context of new efficient and eco-friendly biocidal mixtures. On the other hand, since 2021, a novel multi- surface disinfectant spray based on **APDA** (< 1 wt.%) is marketed by Pritchard Spray Technology Ltd. under the brand name Virusend^TM^ [40]. Initially, developed by the British Army, this spray is claimed to eliminate 99.99% (Δlog_10_ = 4) of bacteria (e.g., *Staphylococcus*, *Escherichia*, *Salmonella*, *Listeria*) and enveloped viruses (e.g., SARS-CoV-2, MPXV, influenza virus, respiratory syncytial virus) in under a minute [42,43]. Moreover, it is claimed that this product is also active against non-enveloped viruses (e.g., rhinovirus, adenovirus, enterovirus and norovirus) [42]. Consequently, our pioneering formulation could have a global impact in the pathogen prevention and change the way we fight current and all future outbreaks. Obviously, between this fundamental research and before use in public, institutional and household spaces, technological development steps remain essential to deliver robust and universal biocides.

## 4. Conclusions

We have demonstrated that **APDA** and **DDAC** associated with **γ-CD** show synergistic action against non-enveloped viruses (PV-1 and MNV) and against bacteria (*P. aeruginosa*). Indeed, this mixture acts as a much more efficient biocide than each compound alone. The virucidal mechanism was ascribed to: (*i*) the protonation of **APDA** leading to pH modification and facilitating the co-micelles dissociation, (*ii*) the partial denaturation of the viral capsid proteins upon pH changes facilitating the passage of **APDA** and **DDAC** through the capsid, (*iii*) the charges screening of the viral nucleic acid, (*iv*) the full dislocation of the viral capsid, and (*v*) the prevention of the protein’s re-aggregation by the **γ-CDs** via chaperone-like activity. On the flip side, the bactericidal action was assigned to: (*i*) the modification of pH upon the protonation of **APDA**, (*ii*) the fast insertion/removal between the cations (**DDAC** and charged **APDA**) and the phospholipids, and (*iii*) the lipid extraction by the **γ-CDs**. Whatever the specific mechanism, the exposure of the genome allows the virus inactivation or cellular death. The tests carried out, according to the European standard EN 14476, demonstrated virucidal efficacy against PV-1 and MNV. As these non-enveloped viruses are highly resistant, when a disinfectant product is effective against them, it can be considered that it will be effective against all types of viruses, including enveloped viruses (SARS-CoV-2 or MPXV). Consequently, “general virucidal activity” can be claimed. In the current pandemic situation, this highly universal disinfectant provides elements valuable to avoid the spread of infectious viral diseases in community settings, including COVID-19 and monkeypox (caused by enveloped viruses). Work is underway to shift to concrete applications, as it can have a global impact on the prevention of known and emerging viruses.

## Figures and Tables

**Table 1:** Surface tension data of the various investigated surfactant systems.

	pH	CMC (mM)	σ^∞^ (mN/m)
**DDAC**	-	1.2	30.0
**APDA**	1	2.5	40.0
	7	2.4	38.0
	12	1.0	37.2
**DDAC/γ-CD**	-	7.5	30.4
**APDA/γ-CD**	1	498	39.9
	12	1.1	37.2
**DDAC/APDA**	12	0.1	34.4
**DDAC/APDA/γ-CD**	12	0.1	32.1

**Table 2:** **DDAC** and **APDA** surface adsorption, and their binding parameters with the **γ-CD**.

Surfactant	pH	Surfactant Adsorption Parameters ^1^			Binding Paramters with γ-CD ^2^		
		Γ_max_ (mol/m^2^)	K_ads_ (M^−1^)	R^2^	Type	K_ass_ (M^−1^)	R^2^
**DDAC**	-	3.04 × 10^−6^	241,000	0.9977	1:1	6,100	0.9980
**APDA**	1	5.16 × 10^−6^	4500	0.9927	1:1	69,000	0.9952
	7	4.67 × 10^−6^	8000	0.9713	n.d.	n.d.	n.d.
	12	4.27 × 10^−6^	28,000	0.9940	1:1	210	0.9964

^1^ Calculated from surface tension data of individual surfactants with Equations (3) and (4). ^2^ Calculated from surface tension data of single surfactant and **γ-CD** (Equations (3), (4), (7) and (8)).

## Data Availability

Not applicable.
